# TMPO-AS1 promotes cell proliferation of thyroid cancer via sponging miR-498 to modulate TMPO

**DOI:** 10.1186/s12935-020-01334-4

**Published:** 2020-07-08

**Authors:** Zhenyu Li, Yun Feng, Zhen Zhang, Xiaozhong Cao, Xiubo Lu

**Affiliations:** 1 Department of Thyroid Surgery, The First Affiliated Hospital of Zhengzhou University, 1 Jianshe East Road, Zhongyuan District, Zhengzhou, 450000 Henan China; 2 Department of Thyroid Surgery, Luoyang Central Hospital Affiliated to Zhengzhou University, Luoyang, 471000 Henan China

**Keywords:** Thyroid cancer, TMPO-AS1, miR-498, TMPO

## Abstract

**Background:**

Thyroid cancer (TC) is the most frequent endocrine malignancy. Long noncoding RNAs (lncRNAs) have been confirmed to act as significant roles in tumor development. The role of lncRNA TMPO-AS1 in TC is still unclear, so it remains to be explored. The aim of the research is to investigate the role and regulatory mechanism of TMPO-AS1 in TC.

**Methods:**

TMPO-AS1 and TMPO expression in TC tumors and cells was detected by TCGA database and QRT-PCR assay respectively. CCK-8, EDU, TUNEL and western blot assays were conducted to identify the biological functions of TMPO-AS1 in TC. Luciferase reporter and RNA pull down assays were conducted to measure the interaction among TMPO-AS1, TMPO and miR-498.

**Results:**

TMPO-AS1 was overexpressed in TC tissues and cell lines. Knockdown of TMPO-AS1 suppressed cell growth and accelerated cell apoptosis in TC. Furthermore, downregulation of TMPO-AS1 suppressed TMPO expression in TC. The data suggested that TMPO expression was upregulated in TC tissues and cell lines and was positively correlated with TMPO-AS1 expression in TC. Furthermore, the expression of miR-498 presented low expression in TC cells. And miR-498 expression was negatively regulated by TMPO-AS1, meanwhile, TMPO expression was negatively regulated by miR-498 in TC cells. Besides, it was confirmed that TMPO-AS1 could bind with miR-498 and TMPO in TC cells. In addition, it was validated that TMPO-AS1 elevated the levels of TMPO via sponging miR-498 in TC cells.

**Conclusions:**

TMPO-AS1 promotes cell proliferation in TC via sponging miR-498 to modulate TMPO.

## Background

Thyroid cancer (TC) is a typical subtype of endocrine malignancy. The incidence and mortality of TC were stably rising over the past decades [1–3]. Although many researches have been made in the diagnosis and treatment, the prognosis in TC patients still faces a severe challenge and was dismal [4, 5]. Thus, exploring underlying molecular therapeutic targets for TC is of great importance to clinical practice.

Long non-coding RNAs (lncRNAs) are a group of non-coding RNAs longer than 200 nucleotides [6, 7]. Previous literature has verified that lncRNAs exerted key roles in the progression of multiple cancers and worked as either oncogenes or tumor suppressors. LncRNAs have been reported to impact biological processes like cell proliferation, apoptosis and metastasis via sponging miRNAs to modulate proteins. For example, lncRNA STCAT16 suppresses cell growth in gastric cancer [8]. LncRNA PEG10 sponges miR-134 to exert its oncogenic function in bladder cancer [9]. Interestingly, lncRNA SNHG7 acts as a sponge of miR-342-3p to promote the occurrence of pancreatic cancer [10]. TMPO-AS1 is a lncRNA that has been reported as a facilitator in various malignant tumors, including prostate cancer [11], cervical cancer [12] and non-small cell lung cancer [13]. Nonetheless, the role and molecular mechanisms of TMPO-AS1 in TC remains to be further explored. This work was aimed at exploring the potential role of TMPO-AS1 in modulating TC cell functions.

LncRNAs with different cellular distribution can regulate their downstream genes through different mechanisms. In the nucleus, lncRNAs can function as protein scaffolds to guide chromatin-modification of their target genes [14–16]. In the cytoplasm, lncRNAs can serve as molecular sponges for miRNAs and modulate the miRNAs’ targets [17, 18]. Mechanistically, lncRNAs have been widely reported as miRNAs’ sponges. Dysregulation of lncRNAs and miRNAs have been reported to be closely associated with the diagnosis of cancers [19–21]. Therefore, exploring novel lncRNAs and their downstream miRNAs is important to finding novel diagnostic biomarkers or therapeutic targets in thyroid cancer. LncRNAs have also been reported as regulators for their antisense mRNAs in human cancers [22, 23]. The focus of our current study was to detect the mechanism by which TMPO-AS1 regulated TMPO and facilitated TC cell growth and migration.

## Methods

### Tissues samples

TC patient tumors and adjacent noncancerous tissues were collected from 40 patients that underwent surgery at the First Affiliated Hospital of Zhengzhou University. None of these enrolled patients undergone any anti-tumor therapy. Following collection, samples were snap frozen and stored at − 80 °C. All patients participated in the present study provided written informed consent, and the Ethics Committee of the First Affiliated Hospital of Zhengzhou University approved this study.

### Cell lines

Human thyroid cancer cell lines (TPC-1, IHH-4, A-PTC and CUTC5) and human normal thyroid epithelial cell line (nthy-ori3-1) were acquired from American Type Culture Collection (ATCC, Manassas, VA). RPMI-1640 medium (Gibco, Rockville, MD) that contains 10% fetal bovine serum (FBS; HyClone, Logan, UT), streptomycin (100 mg/ml) and penicillin (100 U/ml) was used for cell incubation. The incubator was set at 37 °C with 5% CO_2_ in humid air. All cell lines were available according to the STR authentication.

### Quantitative real time PCR (qRT-PCR)

As described previously [19], total RNA was extracted from cultured cells with the application of TRIzol reagent (15596018, Invitrogen, Carlsbad, CA, USA). Synthesis of complementary DNA (cDNA) for miRNAs, mRNAs and lncRNAs was carried out using TaqMan™ MicroRNA Reverse Transcription Kit (4366597, Thermo Fisher Scientific, Waltham, MA, USA) and PrimeScriptTM II Reverse Transcriptase (2690A, Takara, Tokyo, Japan), respectively. qPCR analysis was carried out by SYBR Green PCR Kit (Takara) on ABI7900 system (Applied Biosystems, Foster City, CA). The expression of genes was quantified using 2^−ΔΔCt^ method. Internal control was GAPDH or U6. All primer sequences were provided in Additional file 1: Table S1. Each experimental procedure was repeated at least in triplicate.

### Cell transfection

As described previously [24], the short hairpin RNAs (shRNAs) specific to TMPO-AS1 or TMPO were synthesized by GenePharma (Shanghai, China), and nonspecific shRNAs served as negative control (NC). TMPO sequence was subcloned into pcDNA3.1 (Invitrogen) to produce pcDNA3.1/TMPO, with empty pcDNA3.1 used in NC group. miR-498 mimics, miR-498 inhibitor and negative controls were bought from GenePharma (Shanghai, China). Then plasmids mentioned above were transfected into TPC-1 and IHH-4 cells by Lipofectamine2000 (Invitrogen) in accordance with manufacturer’s suggestions. Sequences for plasmids were provided in Additional file 1: Table S1. Each experimental procedure was repeated at least in triplicate.

Stably transfected cells with TMPO-AS1 silence were constructed for this study. Parental cells were planted in 96 well plates at 37 °C for 12 h. Puromycin was added into the well at the final concentration of 2, 4, 6, 8, 10 μg/ml (each density in 3 well). Change the reagent every 2–3 day. One week later, the lowest concentration at which cells were killed was identified. Next, using the virus to infect the cells. 7–14 days later, the stably transfected cells were selected out for subsequent experiments.

### Cell Counting Kit-8 (CCK-8)

As described previously [25], stably transfected cells (1 × 10^3^) were prepared in 96-well plates. After cell adherence, each well was introduced with 10 μl of CCK-8 solution for 4 h at 37 °C. Cell viability was detected at 0, 24, 48, 72 h by microplate absorbance reader (Bio-Rad Laboratories, Hercules, CA) at 450 nm.

### 5-Ethynyl-2′-deoxyuridine (EdU) assay

As described previously [26], stably transfected cells were added into 24-well plates, fixed with 4% formaldehyde solution in phosphate-buffered saline (PBS; Invitrogen). The proliferation ability of transfected cells was assessed by EdU kit (RiboBio, Guangzhou, China) based on the instruction of the manufacturer. The nuclei double-stained with EdU and 4′-6-diamidino-2-phenylindole (DAPI, Sigma-Aldrich, Miamisburg, OH) were considered to be positively proliferative cells.

### Colony formation assay

As described previously [27], stably transfected TC cells (500 cells in each well) were severally transferred into 6-well plates, grown in complete media containing 10% FBS and grown in complete media containing 10% FBS for 14-day cell culture purposes. After fixation, cells were subjected to 0.5% crystal violet staining, and counted to determine clone formation. The number of colonies was counted by the eyes. Each experimental procedure was repeated at least in triplicate.

### Terminal deoxynucleotidyl transferase dUTP nick end labeling (TUNEL)

As described previously [28], in situ cell death detection kit (Roche, Penzberg, Upper Bavaria, Germany) was utilized to identify the apoptotic cells under the manufacturer’s protocols. Nuclei were stained by DAPI. An inverted laser scanning confocal microscope was utilized for the capture of images. At last, quantification of TUNEL-positive cells was determined through ImageJ software. Each experimental procedure was repeated at least in triplicate.

### Flow cytometry

As described previously [29], cell apoptosis was assessed by flow cytometry after double-staining with Annexin V-fluorescein isothiocyanate (FITC)/propidium iodide (PI) detection kit (Invitrogen) as per guidebook. At length, apoptotic rate of cell lines was assessed by a Cytoflex flow cytometer (Beckman Coulter, Inc.250 S.Kraemer Boulevard Brea, CA 92821, USA). Data were assessed with FlowJo software version 10.5.3 (Tree Star, USA). Each experimental procedure was repeated at least in triplicate.

### Western blot

As described previously [30], samples of TC cells were lysed via RIPA buffer (R0278, Sigma-Aldrich, St. Louis, MO, USA), and the protein concentration was measured through the BCA Protein Assay kit (23227, Pierce Biotechnology, Rockford, IL). Equal amount of proteins was separated via 12% SDS-PAGE (1610174, Bio-Rad Laboratories, Hercules, CA) and transferred to PVDF membranes (IPVH00010, Millipore, Bedford, MA). The membranes were blocked with 5% skim milk and incubated with primary antibodies against loading control GAPDH (ab181602; 1:1000 dilution) and TMPO (ab185718; 1:1000 dilution), as well as the corresponding secondary antibodies (ab6721; 1:2000 dilution). Antibodies used in this study were all obtained from Abcam (Cambridge, MA, USA). ECL detection kit (32134, Pierce Biotechnology, Rockford, IL) was adopted to detect the bands. Each experimental procedure was repeated at least in triplicate.

### Transwell assay

As described previously [31], cell invasion was detected by Matrigel-coated transwell chamber (353097, Corning Co, Corning, NY, USA) as per manufacturer’s instructions. The lower chamber was supplemented with complete medium, and cells in serum-free medium were added to upper chamber. Invading cells were fixed and stained in crystal violet solution for counting. Cell migration assay was performed without Matrigel coating. The number of migrated or invaded cells was measured under a microscope (200 μm, DMI8, Leica, Wetzlar, Germany). Each experimental procedure was repeated at least in triplicate.

### In vivo experiments

Nude male mice (6 weeks old; n = 6) were randomly divided into two groups with equal amounts. As described previously [32], each mouse in two groups was separately subcutaneously injected into 4 × 10^5^ TPC-1 cells stably transfected with sh-TMPO-AS1 or sh-NC. Tumor volume was measured every 4 days according to following formulas: larger diameter × (smaller diameter)^2^/2. After 4 weeks, these animals were then euthanized and the tumors were removed and weighted. Animal study was conducted and approved by the Ethics Committee of the First Affiliated Hospital of Zhengzhou University.

### Bioinformatics analysis

TMPO-AS1 and TMPO expression in TCGA COAD samples and normal samples as well as its expression correlation with TMPO was obtained from GEPIA database (http://gepia2.cancer-pku.cn/#analysis) [33]. Additionally, the expression patterns of TMPO-AS1, miR-498 and TMPO in COAD samples and normal samples were obtained from pan-cancer data in starabse (http://starbase.sysu.edu.cn). Similarly, the expression correlations among these three RNAs were analyzed based on starbase pan-cancer data. The genomic location of TMPO-AS1 and TMPO was obtained from UCSC (http://genome.ucsc.edu/). MiRNAs potentially bind with TMPO-AS1 were predicted using two databases (starbase and DIANA tool: http://carolina.imis.athena-innovation.gr/diana_tools/web/index.php?r=lncbasev2%2Findex-predicted).

### Luciferase reporter assay

Luciferase reporter assay was conducted as described previously [34]. TMPO promoter region was subcloned into pGL3 vectors (Promega, Madison, MI), and co-transfected with sh-NC or sh-TMPO-AS1 to 293T and TPC-1, IHH-4 cells. Besides, TMPO-AS1 or 3′-UTR sequences of TMPO fragments covering miR-498 binding sites (wild-type or mutant) were separately inserted to pmirGLO vectors (Promega), then co-transfected with miR-498 mimics or NC mimics to cells. Luciferase activity was evaluated with luciferase reporter assay system (Promega) after cells were incubated for 48 h. Each experimental procedure was repeated at least in triplicate.

### Fluorescence in situ hybridization analysis (FISH)

TMPO-AS1 FISH probe was produced by RiboBio (Guangzhou, China) based on manufacturer’s protocol, and FISH assay was performed as described previously [35]. Sequence for probe was listed in Additional file 1: Table S1. Cells were first fixed and washed in PBS, then air-dried cells were cultured with FISH probes in hybridization buffer. The slides were washed, dehydrated, and mounted with DAPI. Olympus microscope (Tokyo, Japan) was utilized to capture the image of the slides for immunofluorescence. Each experimental procedure was repeated at least in triplicate.

### Subcellular fractionation assay

PARIS kit (Invitrogen) was applied to isolate cytoplasmic and nuclear fractions from thyroid cancer cells, as described previously [35]. Cells were planted in two wells of six well plates at a density of 2 × 106 cells per well. Remove the medium in six well plates and add into 2 ml 1× PBS to wash cells. Afterwards, the PBS was removed and 200 μl pre-cool lysis buffer J following by the incubation on ice to permeate cells completely. Cell lysis was then removed into a 1.5 ml EP tube without enzyme and centrifuged at 14000 cycle/min for 10 min. The cytoplasmic RNA products were added into 200 μl Buffer SK, while the nuclear RNA products were added into 200 μl Buffer SK. After washing, RNAs were preserved at − 80 °C. The distribution of TMPO-AS1 in cytoplasm or nucleus was determined by qRT-PCR. The reaction condition is 25 °C for 5 min, 42 °C for 60 min, 70 °C for 5 min. GAPDH and U6 were separately served as cytoplasmic control and nuclear control. Each experimental procedure was repeated at least in triplicate.

### RIP assay

RIP assay was conducted through the use of Magna RNA-binding protein immunoprecipitation kit (Millipore, Billerica, MA, USA), as described previously [36]. Cell lysate was prepared in RIP buffer that was supplemented with magnetic beads coated on Ago2 antibody (SAB4200724). IgG antibody (401455-2ML-M, Millipore) acted as control. Immunoprecipitated RNAs were then extracted, qRT-PCR was employed to quantify purified RNAs. Each experimental procedure was repeated at least in triplicate.

### RNA pull-down assay

RNA–Protein Pull-Down Kit (Thermo Fisher Scientific, Waltham, MA) was applied for RNA pull-down assay in accordance with manufacturer’s suggestions, as described previously [37]. The biotinylated RNAs for TMPO-AS1 or miR-498 and control probe were synthesized. At first, the cells were plated in six well-plate and transfected with probes. 48 h later, 1% polyformaldehyde was used for crosslink and glycine was used to terminate the crosslink. Next, cell was lysed and digested with genomic DNA. Streptomyces philophile magnetic beads was used to capture miRNA complex. After washing, proteinase K was added to wipe off the protein and unbind the crosslink. Finally, RNA was collected as previously mentioned and was measured by qRT-PCR under the condition as follows: pre-denaturation at 95 °C for 60 s, denaturation at 95 °C for 60 s, annealing at 58 °C for 60 s. Each experimental procedure was repeated at least in triplicate. Primer sequences used in this experiment were listed in Additional file 1: Table S1.

### Statistical analysis

Statistical analysis was conducted by use of GraphPad Prism 6 (GraphPad Software, Inc., La Jolla, CA). Data was presented as mean ± SD. One-way ANOVA analysis or two-tailed Student’s t-tests were conducted to analyze differences among multiple groups or between groups. P < 0.05 was thought of statistical significance. Each experiment of this study was performed at least three times.

## Results

### The expression of TMPO-AS1 is significantly upregulated in TC tissues and cells and facilitates the functions of TC cells

**Fig. 1:**
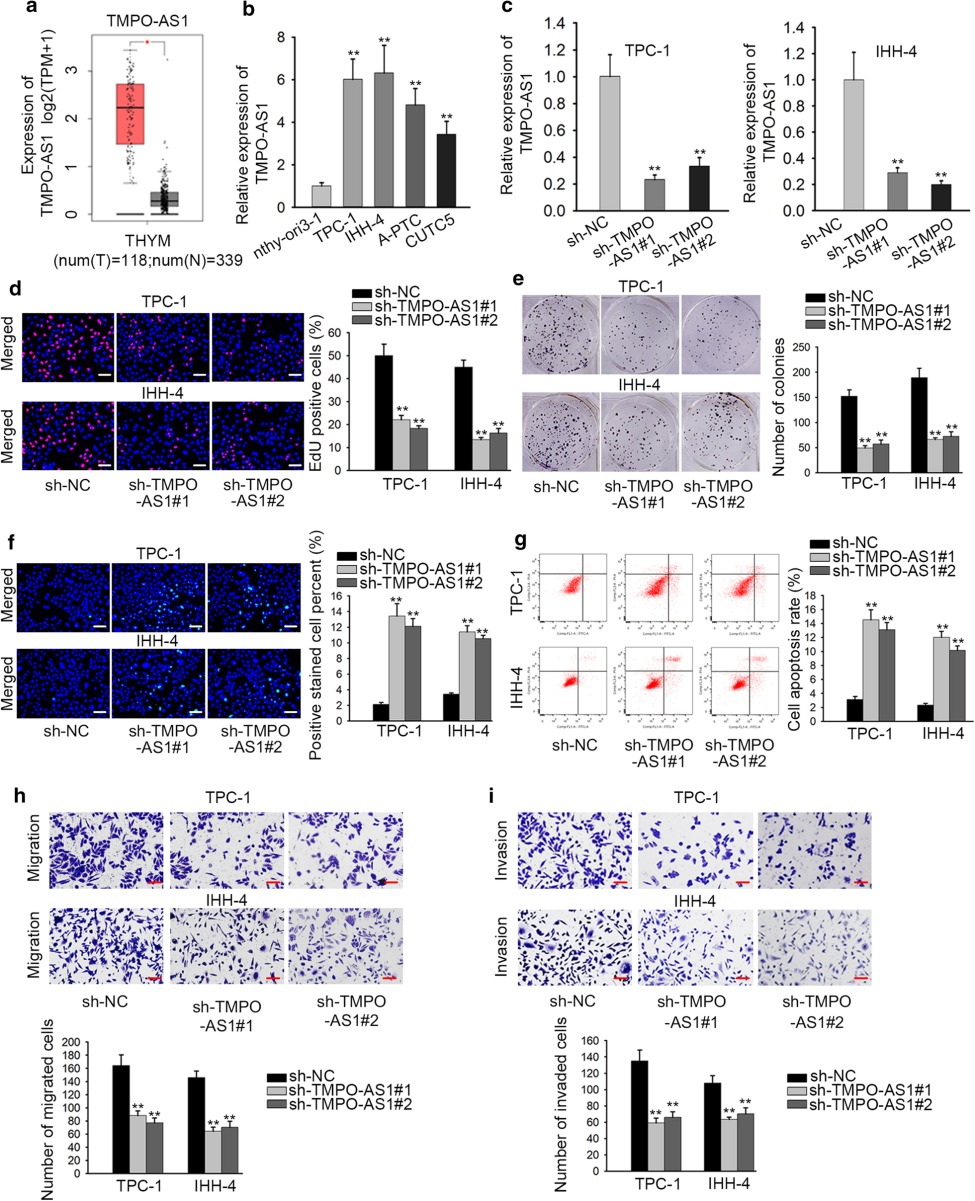
The expression of TMPO-AS1 is significantly upregulated in TC tissues and cells and facilitates TC cellular processes. **a** TMPO-AS1 expression was upregulated in TC tissues from TCGA database. **b** QRT-PCR assay was utilized to detect the expression of TMPO-AS1 in TC cell lines (A-PTC, CUTC5, TPC-1 and IHH-4) and human normal thyroid epithelial cell line (Nthy-ori3-1). **c** The knockdown efficiency of TMPO-AS1 was assessed by QRT-PCR assay in TPC-1 and IHH-4 cells transfected with sh-TMPO-AS1#1/2 or sh-NC. **d**, **e** The proliferation ability of transfected cells was measured by EdU assays and colony formation assay. **f**, **g** TUNEL and cytometry analysis was conducted to evaluate cell apoptosis rate in transfected cells. **h**, **i** Migration and invasion were examined in different groups in transwell assays. **p < 0.01

At first, we obtained the information from GEPIA database and found that TMPO-AS1 was upregulated in TC tissues (Fig. 1a, p < 0.01). Subsequently, the level of TMPO-AS1 was evaluated in four TC cells and human normal thyroid cell line nthy-ori3-1 (which was set as 1.0). As shown in Fig. 1b, the relative level of TMPO-AS1 in four TC cells was separately 6.018, 6.319, 4.8103 and 3.424. Moreover, the TMPO-AS1 was expressed at highest level in TPC-1 and IHH-4 cells, thus we chose them for subsequent experiments. As shown in Fig. 1c, TMPO-AS1 expression was decreased from 1.0 to 0.231 and 0.331 in TPC-1 cells after transfection with sh-TMPO-AS1#1/#2 (p < 0.01). Consistently, the same effect of sh-TMPO-AS1#1/#2 was observed in IHH-4 cells. The results from EdU assay indicated that the percent of EdU positive cells was reduced 27% and 32% in TPC-1 cells, while it was decreased into 13% and 16% in IHH-4 cells (Fig. 1d, p < 0.01). Colony formation assays manifested that TMPO-AS1 depletion led to the decreased number of colonies (Fig. 1d, e, p < 0.01). According to the data obtained by performing TUNEL assays, the percentage of positive cells was increased into 14.52% and 13.11% in TPC-1 cells (Fig. 1f left, p < 0.01). In addition, positive cell percent was increased into 12.01% and 10.14% (Fig. 1f right, p < 0.01). Through flow cytometry analysis, TMPO-AS1 inhibition accelerated the apoptosis of TPC-1 and IHH-4 cells (Fig. 1g, p < 0.01). On the contrary, migration and invasion were repressed by down-regulated TMPO-AS1 in transwell assays (Fig. 1h, i). The number of migrated cells was decreased from 164 into 88 and 77 in TPC-1 cells, and it was reduced into 64 and 70 in IHH-4 cells. In a word, TMPO-AS1 was remarkably high in TC tissues and cell lines, and TMPO-AS1 inhibition restrained growth, migration and invasion in TC cells.

### TMPO-AS1 positively regulates its nearby gene TMPO in TC

**Fig. 2:**
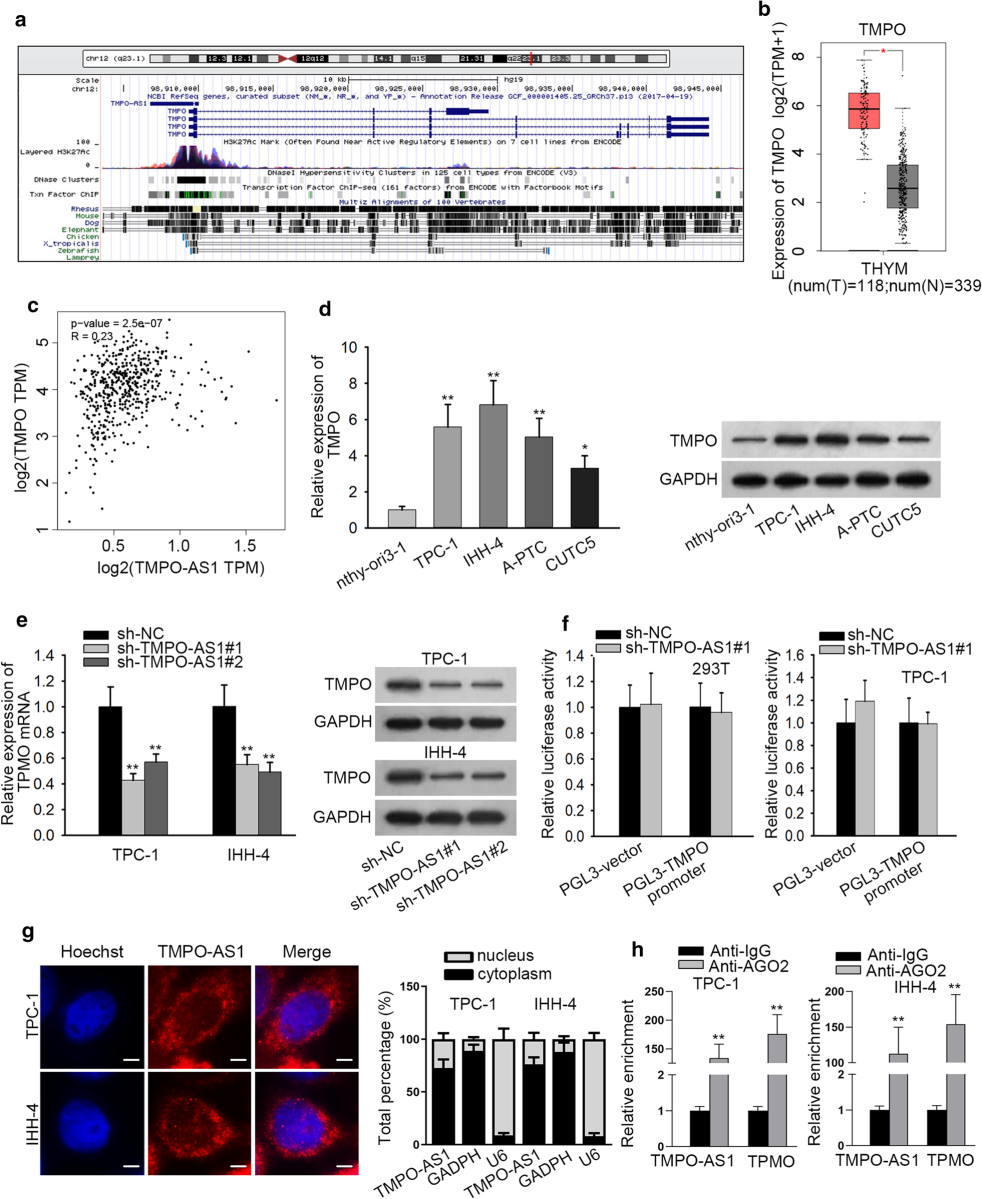
TMPO-AS1 positively regulates TMPO in TC. **a** The location of TMPO-AS1 in chromatin was displayed. **b** TMPO was upregulated in TC tissues from TCGA database. **c** There was a positive correlation between TMPO-AS1 and TMPO from GEPIA database. **d** The expression of TMPO in TC cell lines and human normal thyroid epithelial cell line was detected by QRT-PCR. **e** QRT-PCR was performed to examine the mRNA expression of TMPO in transfected cells. **f** Luciferase reporter assay was used to detect the interaction between TMPO-AS1 and TMPO promoter. **g** It was tested by FISH and subcellular fractionation assays that TMPO-AS1 mainly located in cytoplasm. **h** RIP assays demonstrated TMPO-AS1 and TMPO were enriched in Ago2 antibody. *p < 0.05, **p < 0.01

Searching from UCSC database, we determined that TMPO was the nearby gene of TMPO-AS1 in genome (Fig. 2a). According to the GEPIA database, the expression of TMPO was dramatically upregulated in TCGA TC tissues (Fig. 2b). Besides, TMPO-AS1 expression had positive correlation with TMPO expression in TCGA TC tissues (Fig. 2c). Subsequently, TMPO expression was measured in different cells. When TMPO level in nthy-ori3-1 was set as 1.0, the level in TPC-1, IHH-4, A-PTC and CUTC5 cells was separately 5.58, 6.81, 5.03, 3.30 (Fig. 2d, p < 0.05, p < 0.01), indicating that TMPO had a high level in four TC cell lines. Similarly, the protein level of TMPO was increased in TC cells compared with normal cell. More importantly, the mRNA level of TMPO were decreased about 50% by the knockdown of TMPO-AS1 in TPC-1 and IHH-4 cells (Fig. 2e, p < 0.01). This tendency was further reflected in the protein level of TMPO. To explore the effects of TMPO-AS1 on TMPO transcription, luciferase reporter assays were carried out in 293T and TPC-1 cells transfected with PGL3-TMPO-AS1 promoter or PGL3-vector, together with sh-NC or sh-TMPO-AS1#1. No notable difference was observed in the luciferase activity of TMPO promoter reporter between the two groups of differently transfected cells (Fig. 2f, p > 0.05). Besides, subcellular localization condition and FISH assay revealed that TMPO-AS1 was localized mainly in the cytoplasm of TPC-1 and IHH-4 cells (Fig. 2g). Meanwhile, the results of RIP assays exhibited that TMPO-AS1 and TMPO were enriched in RISC (Fig. 2h). Taken together, TMPO-AS1 positively modulated TMPO in TC cells.

### MiR-498 is downregulated and negatively regulated by TMPO-AS1 as well as represses the cellular process in TC

**Fig. 3:**
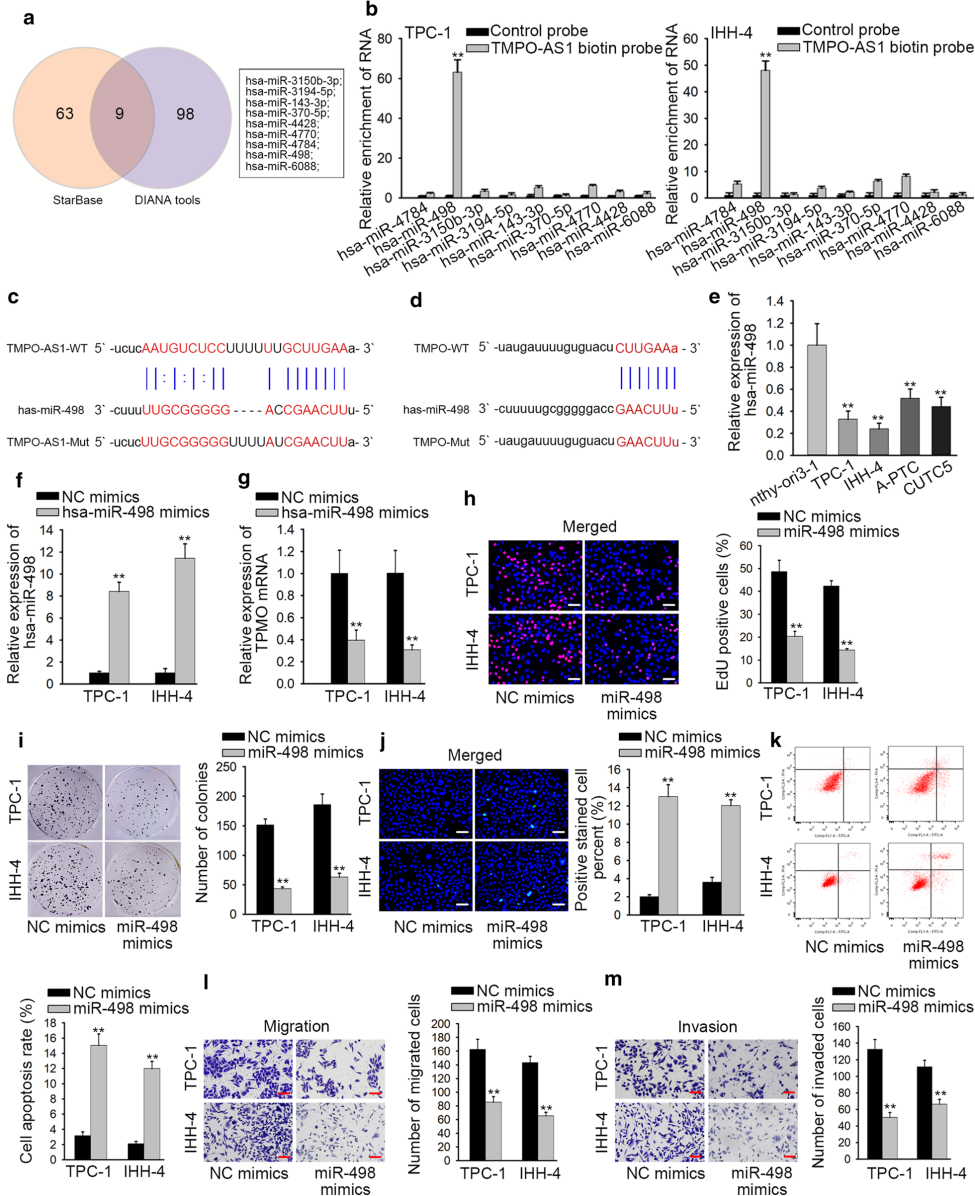
MiR-498 is downregulated and negatively regulated by TMPO-AS1 as well as represses the growth of TC. **a** Target genes that could bind with TMPO-AS1 were obtained from starBase and DIANA tools database. **b** RNA pull down assays selected out miRNAs to bind with TMPO-AS1. **c** MiR-498 had a binding site for TMPO-AS1. **d** MiR-498 had a binding site for TMPO. **e** The expression of miR-498 in TC cell lines and human normal thyroid epithelial cell line was detected by QRT-PCR. **f** QRT-PCR was used to examine the expression of TMPO in TPC-1 and IHH-4 cells transfected with NC mimics or miR-498 mimics. **g** QRT-PCR assays were applied to measure the mRNA expression of TMPO in transfected cells. **h**–**m** The effects of miR-498 on TC growth were assessed by functional assays. **p < 0.01

Considering the enrichment of TMPO-AS1 and TMPO in RISC, we further analyzed whether they could interact with a same miRNA to exert functions. Firstly, we obtained 9 candidate miRNAs (miR-3150b-3p, miR-3194-5p, miR-143-3p, miR-370-5p, miR-4428, miR-4770, miR-4784, miR-498 and miR-6088) that bind to TMPO-AS1 from starBase v2.0 database (containing 72 relevant miRNAs) and DIANA tools database (possessing 107 relevant miRNAs) (Fig. 3a). Additionally, we discovered that miR-498 had the potential to bind with TMPO-AS1 through RNA pull down assays (Fig. 3b, p < 0.01), and the binding sites between miR-498 and TMPO-AS1 were exhibited in Fig. 3c. Then we uncovered and displayed the binding sites between miR-498 and TMPO (Fig. 3d). Additionally, the expression level of miR-498 was lower in TC cell lines than that in nthy-ori3-1 cell (Fig. 3e, p < 0.01). To determine the functions of miR-498 in TC cells, we overexpressed it and conducted gain-of function assays (Fig. 3f, p < 0.01). QRT-PCR analysis demonstrated that miR-498 overexpression suppressed the mRNA level of TMPO (Fig. 3g, p < 0.01). Afterwards, the positive cells were increased in response to the upregulation of miR-498 (Fig. 3h, p < 0.01). The number of colonies was also reduced a lot after overexpression of miR-498 (Fig. 3i, p < 0.01). Meanwhile, the apoptosis was TC cells was measured in miR-498-upregulated cells. Through TUNEL assay and flow cytometry analysis, the apoptosis rate in cells transfected with miR-498 mimics was increased significantly (Fig. 3j, k, p < 0.01). In transwell assays, migratory and invaded capacities were suppressed by up-regulated miR-498 (Fig. 3l, m, p < 0.01). To sum up, miR-498 expression negatively modulated TMPO and served as tumor inhibitor in TC cells.

### TMPO facilitates the biological functions of TC cells

**Fig. 4:**
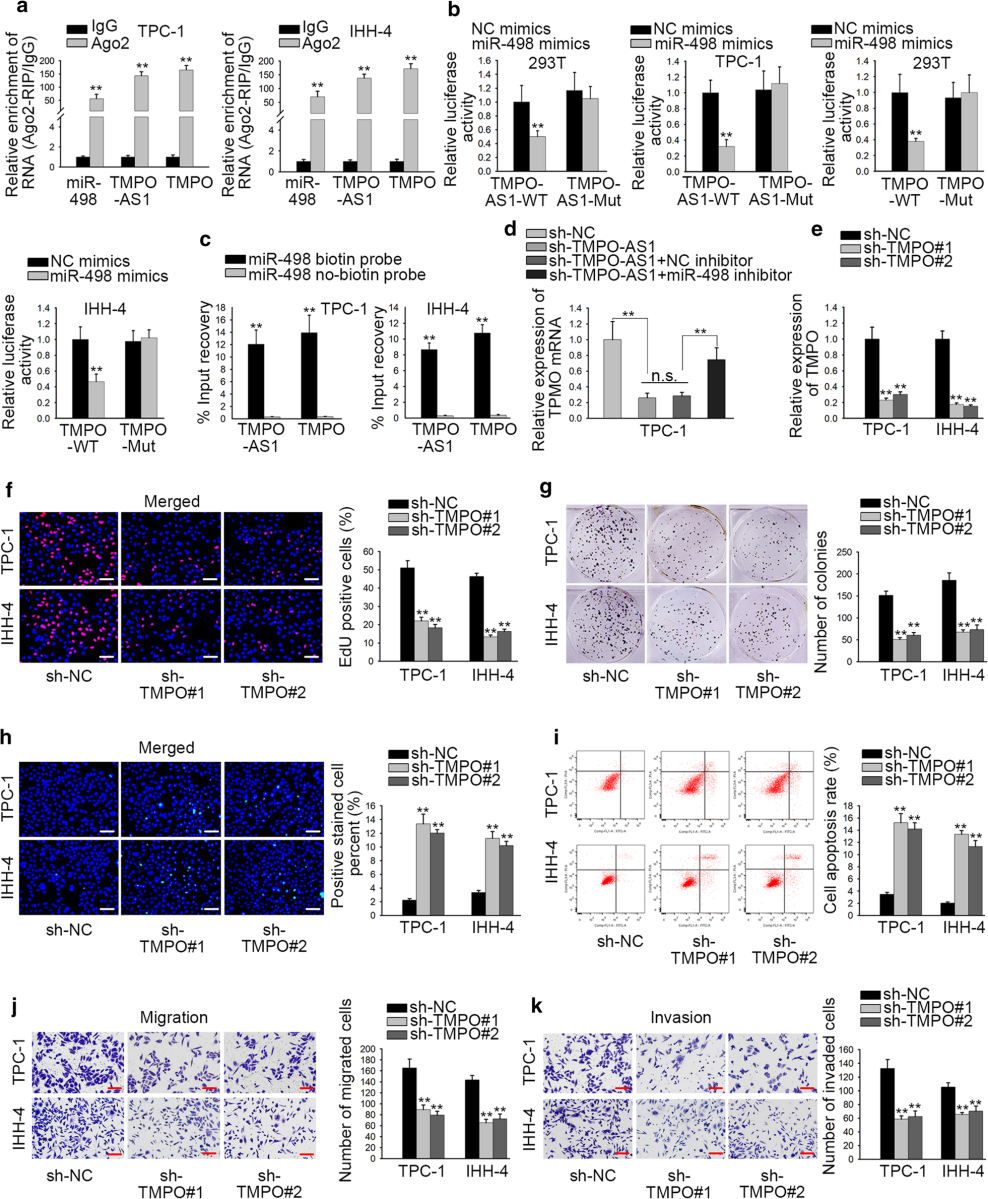
TMPO facilitates the biological functions of TC cells. **a** The interaction among miR-498, TMPO-AS1 and TMPO was testified by RIP assay. **b** Luciferase reporter assay was used to confirm that miR-498 bound with TMPO-AS1 or TMPO. **c** RNA pull-down assay was utilized to validate that miR-498 could bind with TMPO-AS1 or TMPO. **d** The mRNA expression of TMPO was detected by QRT-PCR in transfected cells. **e** The efficiency of sh-TMPO#1/2 was appraised by QRT-PCR. **f**–**k** Functional assays evaluated the influence of TMPO. **p < 0.01. n.s.: no significance

To validate whether TMPO-AS1 accelerated TMPO expression via sponging miR-498 in TC, first, RIP assay verified that miR-498, TMPO, and TMPO-AS1 were co-existed in RISC (Fig. 4a, p < 0.01). Next, luciferase reporter assays results exhibited that miR-498 up-regulation could decrease the luciferase activity of TMPO-AS1-WT and TMPO-WT but failed to affect that of TMPO-AS1-Mut and TMPO-Mut (Fig. 4b, p < 0.01). In addition, RNA pull down assay further testified that miR-498 could bind with both TMPO-AS1 and TMPO (Fig. 4c, p < 0.01). Following the rescue experiments, miR-498 suppression partially rescued the TMPO-AS1 knockdown-mediated inhibition of TMPO mRNA in TPC-1 cell (Fig. 4d, p < 0.01, n.s.: p > 0.05). Additionally, TMPO was silenced in TPC-1 and IHH-4 cells for loss-of function assays (Fig. 4e, p < 0.01). Results displayed that TMPO knockdown had significantly inhibitory effects on proliferation (Fig. 4f, g, p < 0.01) but facilitated apoptosis (Fig. 4h, i, p < 0.01). Moreover, the number of migrated and invaded cells was depleted after silencing of TMPO (Fig. 4j, k, p < 0.01). To summarize, above findings showed that TMPO-AS1 modulated TMPO expression and promoted TC progression via sponging miR-498.

### TMPO-AS1 contributes to the progression of TC via sponging miR-498 to regulate TMPO

**Fig. 5:**
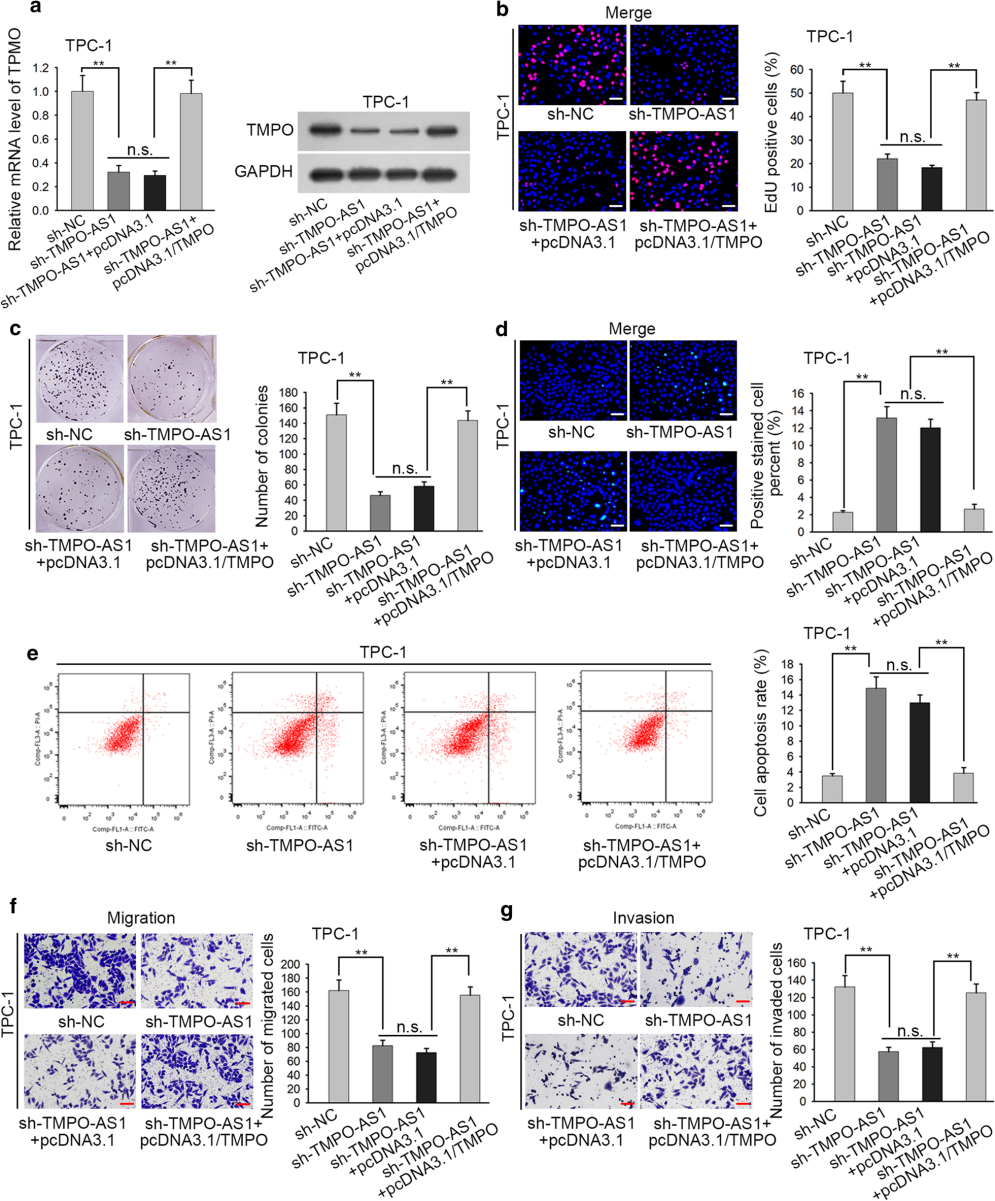
TMPO-AS1 contributes to the progression of TC via sponging miR-498 to upregulate TMPO. **a** TMPO expression was assessed by QRT-PCR in cells with pcDNA3.1/TMPO. **b**, **c** EdU assays and colony formation assays were used to evaluate the proliferative ability in transfected cells. **d**, **e** The apoptosis of transfected cells was measured by TUNEL assay and flow cytometry analysis. **f**, **g** Migration and invasion were appraised in cells transfected with sh-TMPO-AS1#1. **p < 0.01. n.s.: no significance

Finally, we conducted rescue assays to demonstrate the role of ceRNA pathway validated above in TC cells. As the beginning, the levels of TMPO mRNA and protein were examined and the results showed that both levels deceased by silenced TMPO-AS1 were recovered again after overexpressed TMPO (Fig. 5a, p < 0.01, n.s.: p > 0.05). Through proliferation assays, we discovered that TMPO overexpression countervailed the suppressive effects of TMPO-AS1 deficiency on TPC-1 cell proliferation through EdU assays and colony formation assays (Fig. 5b, c, p < 0.01, n.s.: p > 0.05). Furthermore, TMPO up-regulation partially rescued the ascending trend of TMPO-AS1 knockdown on TPC-1 cell apoptosis via TUNEL and flow cytometry analysis (Fig. 5d, e, p < 0.01, n.s.: p > 0.05). In contrast, the falling tendency of migration and invasion induced by down-regulated TMPO-AS1 was reversed by the overexpression of TMPO in transwell assays (Fig. 5f, g, p < 0.01, n.s.: p > 0.05). In conclusion, these findings illustrated that TMPO overexpression could recover the impacts of TMPO-AS1 silence on TC progression.

### TMPO-AS1 promotes TC cell growth in vivo

**Fig. 6:**
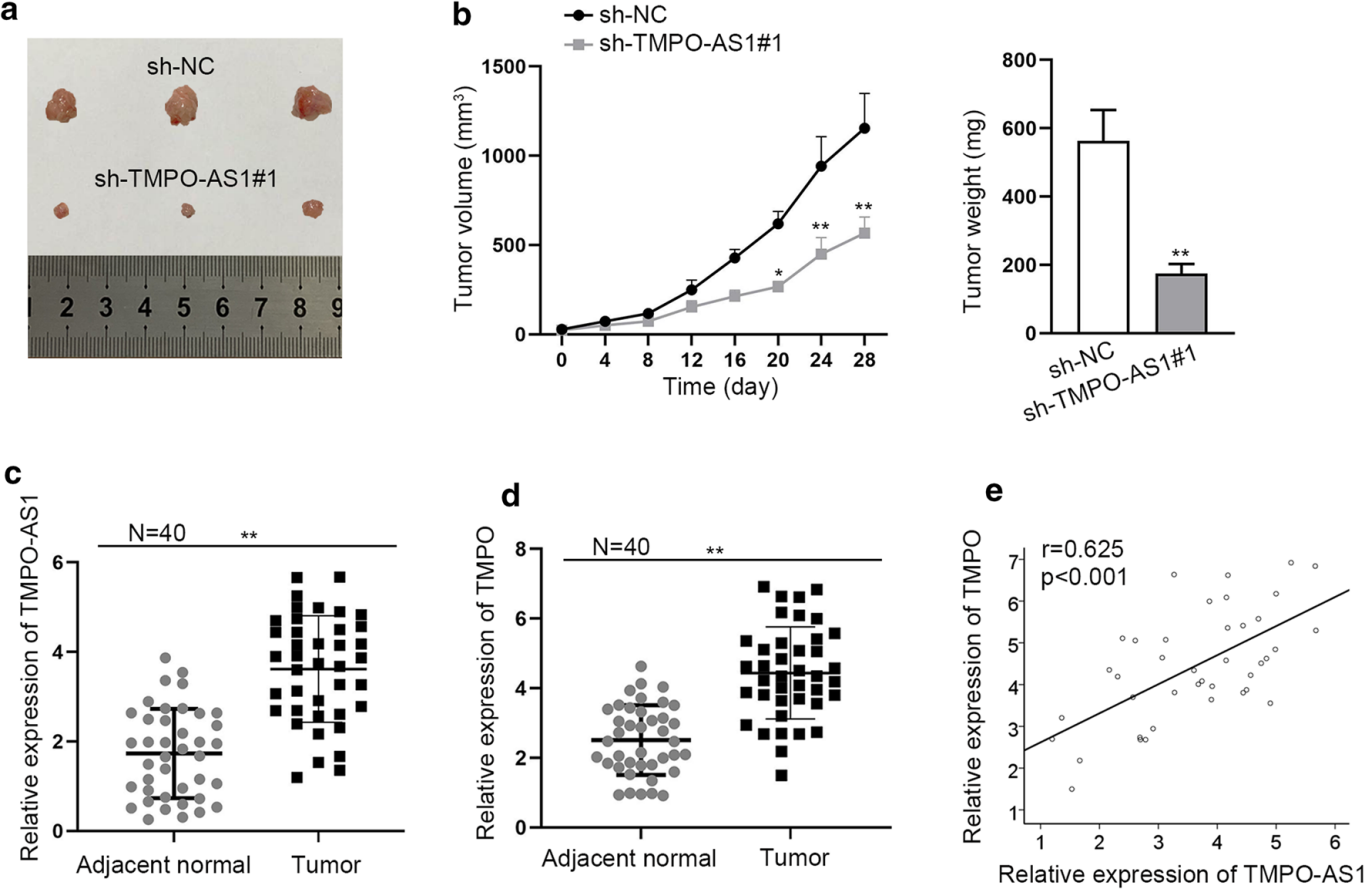
TMPO-AS1 promotes TC cell growth in vivo. **a** Tumors resected from mice injected with TPC-1 cells stably transfected with sh-NC or sh-TMPO-AS1#1. **b** Tumor volume and weigh in two different groups were shown. **c** TMPO-AS1 expression in 40 pairs of TC tissues and adjacent normal tissues. **d** TMPO expression in paired tissues collected from 40 TC patients. **e** Pearson correlation test about TMPO-AS1 and TMPO expression in TC tissues. *p < 0.05, **p < 0.01

In vivo assay was further used to validate above findings. The tumor growth in sh-TMPO-AS1#1 group was found to be slower than that in sh-NC group, which was reflected by the tumor volume and weight (Fig. 6a, b, p < 0.05, p < 0.01). Moreover, TMPO-AS1 was upregulated in TC tissues compared to adjacent normal tissues (Fig. 6c, p < 0.01). Furthermore, high expression of TMPO was also observed in 40 collected TC samples (Fig. 6d), which presented the same tendency with TMPO-AS1 (Fig. 6e). Therefore, we determined that TMPO-AS1 was upregulated in TC patient samples and promoted TC cell growth in vivo.

## Discussion

Plenty of evidence highlighted that lncRNAs exerted essential roles in various human cancers, such as hepatocellular carcinoma [38], non-small cell lung cancer [39], and epithelial ovarian cancer [40]. It has been testified that lncRNA TMPO-AS1 was an oncogene in prostate cancer [11]. In current work, it was demonstrated that TMPO-AS1 was conspicuously overexpressed in TC tissues and cell lines. Besides, depletion of TMPO-AS1 inhibited cell proliferative, migratory and invaded abilities and boosted cell apoptosis. LncRNAs can exert functions in human cancers by regulating their antisense mRNAs. For example, down-regulation of lncRNA ZEB1-AS1 suppresses the proliferation and invasion in OSCC by inhibiting ZEB1 [41]. LncRNA SOX9-AS1 positively regulates SOX9 in hepatocellular carcinoma and drives tumor growth [42]. Here, we examined the level of TMPO mRNA in cells transfected with TMPO-AS1-specific shRNAs and identified that TMPO-AS1 positively regulated TMPO in TC cells. According to the previous researches, TMPO has been reported as an oncogene in lung cancer [43], breast cancer [44], glioblastoma [45] and so on. In our study, TMPO was examined to be upregulated in TC tissues. In addition, there was a positive correlation between TMPO-AS1 and TMPO from GEPIA database. Functionally, silencing of TMPO also exerted the inhibitory effects on the growth and migration of TC cells. Taken together, TMPO-AS1 exerted oncogenic function in TC and its expression positively correlated with that of TMPO.

MicroRNAs (miRNAs) are a class of noncoding RNA about 18 to 25 nucleotides in length and have been verified to participate in tumorigenesis and tumor progression [46, 47]. Increasing evidence showed that lncRNA could act as a sponge for specific miRNA to modulate the occurrence of cancers. For instance, lncRNA LINC00662 became a sponge of miR-34a to exert its oncogenic function in prostate cancer [48]. LncRNA LINC-PINT acted as a tumor suppressor in osteosarcoma through repressing miRNA-21 [49]. Up-regulated lncRNA XIST facilitated cell cycle and EMT process in cervical cancer via reducing miR-140-5p [50]. Here, we also investigated that TMPO-AS1 could act as a ceRNA by sponging miR-498 which has been confirmed to be a tumor suppressor in many cancers [51–54]. Further exploration in this study suggested that miR-498 was remarkably downregulated in TC cells. MiR-498 could bind with TMPO-AS1 and TMPO and negatively regulated TMPO expression. Consistent with previous studies, miR-498 exerted suppressive functions in TC cells. LncRNAs have been reported to sponge miRNAs to modulate mRNA. For example, lncRNA LINC00460 sponged miR-302c-5p and targeted FOXA1 to accelerate progression of lung adenocarcinoma [55]. LncRNA MAFG-AS1 served as a tumor promoter in colorectal cancer through miR-147b/NDUFA4 axis [56]. In this study, we discovered that TMPO-AS1 promoted the expression of TMPO via sponging miR-498 in TC. In addition, TMPO contributed to the cellular processes in TC. Finally, rescue experiments revealed that overexpression of TMPO reversed the suppressive effects of TMPO-AS1 knockdown on TC progression. TMPO-AS1/TMPO axis was also verified in TC clinical samples and animal models. This study revealed the functions of TMPO-AS1 in TC and uncovered a novel ceRNA pathway in TC. All our findings might be helpful for the exploration of novel therapeutic targets. There was also a limitation existed in current study. The upstream molecular mechanism led to the upregulation of TMPO-AS1 remains unclear. We will investigate the upstream molecular mechanism of TMPO-AS1 in future study.

## Conclusion

TMPO-AS1 promoted TC cell growth and migration. TMPO-AS1 exerted functions by sponging miR-498 to upregulate TMPO. TMPO-AS1 might be a novel therapeutic target for TC in the future (Additional files 2, 3, 4, 5, 6).

## Supplementary information

**Additional file 1: Table S1.** The specific primers or sequences used for qRT-PCR, cell transfection, RNA pull down and RNA FISH assays.

**Additional file 2.** Expression profile of miR-498 in thyroid cancer from starBase database.

**Additional file 3.** Expression pattern of TMPO in thyroid cancer from TCGA and starBase database.

**Additional file 4.** The linear correlation analysis between miR-498 and TMPO-AS1 in thyroid cancer was predicted by starbase database.

**Additional file 5.** The linear correlation analysis between miR-498 and TMPO in thyroid cancer was predicted by starbase database.

**Additional file 6.** The linear correlation analysis between TMPO-AS1 and TMPO in thyroid cancer was predicted by starbase database.

## Data Availability

Not applicable.

## Abbreviations

TC: Thyroid cancer
lncRNAs: Long noncoding RNAs
qRT-PCR: Quantitative real time PCR
CCK-8: Cell Counting Kit-8
EdU: 5-Ethynyl-2′-deoxyuridine
TUNEL: Terminal deoxynucleotidyl transferase dUTP nick end labeling
FISH: Fluorescence in situ hybridization analysis
RIP: RNA immunoprecipitation
miRNAs: MicroRNAs
