# Supplementary figures and images for: TMPO-AS1 promotes cell proliferation of thyroid cancer via sponging miR-498 to modulate TMPO
Source: Cancer Cell Int. 2020 Jul 8;20:294. doi: 10.1186/s12935-020-01334-4 (PMC7346673; doi:10.1186/s12935-020-01334-4)

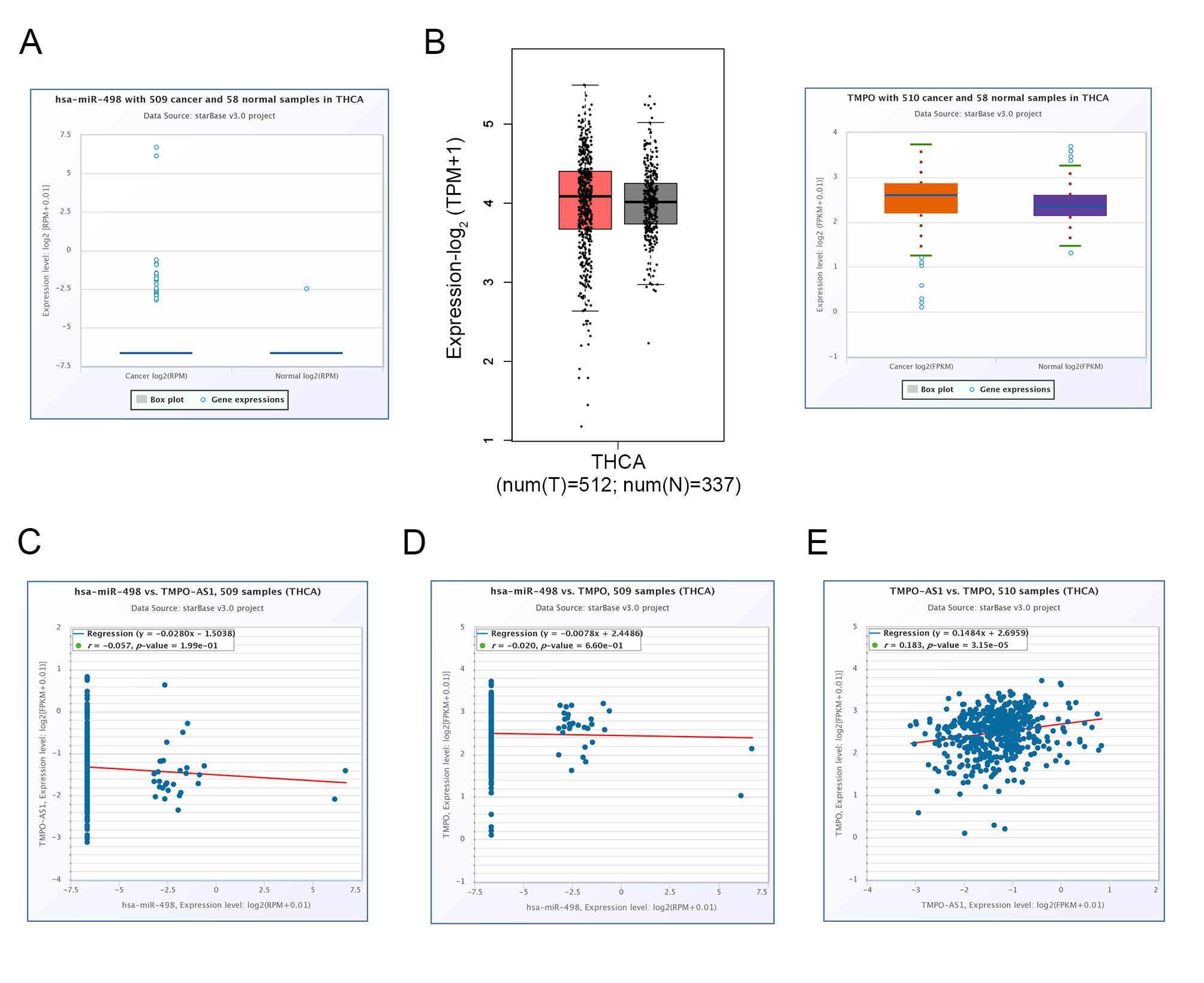

Supplement: Supplementary file 2 — Additional file 2. Expression profile of miR-498 in thyroid cancer from starBase database. [file 12935_2020_1334_MOESM2_ESM.tif]
