# Supplementary material for: TMPO-AS1 promotes cell proliferation of thyroid cancer via sponging miR-498 to modulate TMPO
Source: Cancer Cell Int. 2020 Jul 8;20:294. doi: 10.1186/s12935-020-01334-4 (PMC7346673; doi:10.1186/s12935-020-01334-4)
Supplement: Supplementary file 4 — Additional file 4. The linear correlation analysis between miR-498 and TMPO-AS1 in thyroid cancer was predicted by starbase database. [file 12935_2020_1334_MOESM4_ESM.pdf]

Cell Line Authentication Service

STR Profiling Report

Sample From: the First Affiliated Hospital of Zhengzhou University

Sample Type: Cell Line

Testing Method: STR Genotyping

Report Time: 3/30/2020

Add: Room 502, NO.2 Zhongxing Creative Park, Lane 1015, LongtengRoad, Songjiang District, Shanghai/ Tel:+86-021-33559493
E-mail: market@biowing.com

COMPANY STATEMENT

1. THIS REPORT IS ONLY RESPONSIBLE FOR THE SAMPLES ANALYZED.
2. THE TESTING RESULTS AND THE ORGANIZATION NAME WILL NOT BE USED FOR ADVERTISEMENT, COMMERCIAL EXHIBITIONS, COMMERCIAL PERFORMANCE AND OTHER COMMERCIAL ACTIVITIES.
3. OBJECTIONS SHOULD BE RAISED WITHIN FIFTEEN DAYS AFTER THE RECEIPT OF THIS REPORT.
4. THE PAPER REPORT WITH CONTENT ALTERING, ADDING OR WITHOUT THE STAMPED SEAL OF THE COMPANY ARE INVALID.

Testing Company: Shanghai Chenying Biotechnology Co. Ltd

Address: Room 502, NO.2 Zhongxing Creative Park, Lane 1015, LongtengRoad, Songjiang District, Shanghai

Tel: +86-021-33559493

Contact: Wenyao Zhang

E-mail: market@biowing.com

Cell Line Authentication – STR Profiling Report

Sample code

Table 1. Sample Code

Customer's code	Company Code
IHH-4	20200324-04

Sample Number:1

Sample Type: Cell line

Testing Type: STR

Testing Method:

DNA was extracted by a commercial kit from CORNING (AP-EMN-BL-GDNA-250G). The twenty STRs including Amelogenin locus were amplified by six multiplex PCR and separated on ABI 3730XL Genetic Analyzer. The signals were then analyzed by the software GeneMapper.

Data Interpretation:

Cell lines were authenticated using Short Tandem Repeat (STR) analysis asdescribed in 2012 in ANSI Standard (ASN-0002) by the ATCC Standards Development Organization (SDO) and in Capes-Davis et al., Match criteria for human cell line authentication: Where do we draw the line? Int J Cancer.2013;132(11):2510-9.

Add: Room 502, NO.2 Zhongxing Creative Park, Lane 1015, LongtengRoad, Songjiang District, Shanghai/ Tel:+86-021-33559493
E-mail: market@biowing.com

Test Results

1. STR profile

Table 2. STR and Amelogenin Genotyping Results of Cell line.

Loci	**Sample information**			**Cell Bank information**		
	Sample name：IHH-4			Cell line name：IHH-4		
	**Allele1**	**Allele2**	**Allele3**	**Allele1**	**Allele2**	**Allele3**
D5S818	9	13		9	13	
D13S317	10	13		10	13	
D7S820	9	10		9	10	
D16S539	9	11		9	11	
VWA	16	18		16	18	
TH01	9	9		9	9	
AMEL	X	Y		X	Y	
TPOX	11	11		11	11	
CSF1PO	12	12		12	12	
FGA	23	24				
D21S11	30	31				
D18S51	13	15				
D8S1179	11	15				
D3S1358	16	16				

Add: Room 502, NO.2 Zhongxing Creative Park, Lane 1015, LongtengRoad, Songjiang District, Shanghai/ Tel:+86-021-33559493
E-mail: market@biowing.com

2. database annotation

Figure 1. STR matching analysis

**Cell line name**	IHH-4
**Synonyms**	IHH4
**Accession**	CVCL_2960
**Resource Identification Initiative**	To cite this cell line use: IHH-4 (RRID:CVCL_2960)
**Comments**	Part of: Cancer Cell Line Encyclopedia (CCLE) project.<br>Part of: COSMIC cell lines project.<br>Doubling time: 42 hours (PubMed=11686581).<br>Microsatellite instability: Stable (MSS) (Sanger).<br>Omics: Deep exome analysis.<br>Omics: DNA methylation analysis.<br>Omics: SNP array analysis.<br>Omics: Transcriptome analysis.
**Sequence variations**	AKT1 p.Glu17Lys (c.49G>A) (PubMed=23833040; PubMed=30737244).<br>BRAF p.Val600Glu (c.1799T>A) (PubMed=23833040; PubMed=30737244).<br>CREBBP p.Trp592Ter (c.1776G>A) (PubMed=30737244).<br>CRLF2 p.Trp255Ter (c.765G>A) (PubMed=30737244).<br>EP300 p.Arg1312Ter (c.3934C>T) (PubMed=30737244).<br>RAC1 p.Asp11Glu (c.33C>G) (PubMed=30737244).<br>Heterozygous for TERT c.228C>T (-124C>T); in promoter (PubMed=23833040; PubMed=30737244).
**Genome ancestry**	**Source:** PubMed=30894373 (see ancestry table below)
**Disease**	Thyroid gland papillary carcinoma (NCIt: C4035)<br>Differentiated thyroid carcinoma (ORDO: Orphanet_146)<br>Derived from metastatic site: Left cervical lymph node.
**Species of origin**	Homo sapiens (Human) (NCBI Taxonomy: 9606)
**Hierarchy**	Children:<br>CVCL_3219 (TMH-1)
**Sex of cell**	Male
**Age at sampling**	75Y
**Category**	Cancer cell line
**STR profile**	**Source(s):** Cosmic-CLP; JCRB; PubMed=30737244<br>**Markers:** (see STR table below)

Genome ancestry:

Origin	% genome
African	1.71
Native American	0
East Asian, North	73.08
East Asian, South	21.44
South Asian	0
European, North	0.89
European, South	2.88

STR profile markers:

Amelogenin	X,Y
CSF1PO	12
D3S1358	16
D5S818	9,13
D7S820	9,10
D8S1179	11,15
D13S317	10,13
D16S539	9,11
D18S51	13,15
D21S11	30,31
FGA	23,24
TH01	9
TPOX	11
vWA	16,18

Note: The STR online match analysis of the test cell against EXPASY database, showing cell number (Cell No.) and cell name.

3. Authentication

☐ The submitted sample profile is human, but not a match for any profile in the DSMZ STR database.

☑ The submitted profile is an exact match for the following human cell line(s) in the EXPASY STR database (8 core loci plus Amelogenin): **IHH-4**.

☐ The submitted profile is similar to the following DSMZ human cell line: /.

- **Note:** A cell line can be considered to be authenticated when 80% (exact match) of the alleles in its STR profile match profiles from tissue or other cell line samples from that donor or from database. Cell lines with between a 55% to 80% (similar) match require further profiling for investigation of relatedness.

Add: Room 502, NO.2 Zhongxing Creative Park, Lane 1015, LongtengRoad, Songjiang District, Shanghai/ Tel:+86-021-33559493
E-mail: market@biowing.com

Appendix:

1. Genotyping Strategy and Site Distribution

Table S1. Experimental Strategy and Sites

	Strategy 1	Strategy 2	Strategy 3	Strategy 4
1	D3S1358	D8S1179	D19S433	AMEL
2	VWA	D21S11	TH01	D1S1656
3	D7S820	D16S539	D13S317	D5S818
4	CSF1PO	D2S1338	TPOX	D12S391
5	PENTAE	PENTAD	D18S51	FGA
6			D6S1043	

The allele match algorithm compares the 8 core loci plus amelogenin only, even though alleles from all lociwill be reported when available.

2. DSMZ tools was used to carry on the cell line comparison, which contains 2455 cell lines STR data from ATCC, DSMZ, JCRB ,ECACC， GNE and RIKEN databases. If the cell is not included in the above cell library, users need to compared with other databases.

Technician: Jianan Zhang

Checked by: Ning Qian

Issued by: Yang Bai

Issue date: 3/30/2020

Add: Room 502, NO.2 Zhongxing Creative Park, Lane 1015, LongtengRoad, Songjiang District, Shanghai/ Tel:+86-021-33559493
E-mail: market@biowing.com
