# Supplementary material for: TMPO-AS1 promotes cell proliferation of thyroid cancer via sponging miR-498 to modulate TMPO
Source: Cancer Cell Int. 2020 Jul 8;20:294. doi: 10.1186/s12935-020-01334-4 (PMC7346673; doi:10.1186/s12935-020-01334-4)
Supplement: Supplementary file 6 — Additional file 6. The linear correlation analysis between TMPO-AS1 and TMPO in thyroid cancer was predicted by starbase database. [file 12935_2020_1334_MOESM6_ESM.pdf]

Cell Line Authentication Service

STR Profiling Report

Sample From: the First Affiliated Hospital of Zhengzhou University

Sample Type: Cell Line

Testing Method: STR Genotyping

Report Time: 3/30/2020

COMPANY STATEMENT

1. THIS REPORT IS ONLY RESPONSIBLE FOR THE SAMPLES ANALYZED.
2. THE TESTING RESULTS AND THE ORGANIZATION NAME WILL NOT BE USED FOR ADVERTISEMENT, COMMERCIAL EXHIBITIONS, COMMERCIAL PERFORMANCE AND OTHER COMMERCIAL ACTIVITIES.
3. OBJECTIONS SHOULD BE RAISED WITHIN FIFTEEN DAYS AFTER THE RECEIPT OF THIS REPORT.
4. THE PAPER REPORT WITH CONTENT ALTERING, ADDING OR WITHOUT THE STAMPED SEAL OF THE COMPANY ARE INVALID.

Testing Company: Shanghai Chenying Biotechnology Co. Ltd
Address: Room 502, NO.2 Zhongxing Creative Park, Lane 1015, LongtengRoad, Songjiang District, Shanghai
Tel: +86-021-33559493
Contact: Wenyao Zhang
E-mail: market@biowing.com

Cell Line Authentication – STR Profiling Report

Sample code

Table 1. Sample Code

Customer's code	Company Code
TPC-1	20200324-02

Sample Number:1

Sample Type: Cell line

Testing Type: STR

Testing Method:

DNA was extracted by a commercial kit from CORNING (AP-EMN-BL-GDNA-250G). The twenty STRs including Amelogenin locus were amplified by six multiplex PCR and separated on ABI 3730XL Genetic Analyzer. The signals were then analyzed by the software GeneMapper.

Data Interpretation:

Cell lines were authenticated using Short Tandem Repeat (STR) analysis asdescribed in 2012 in ANSI Standard (ASN-0002) by the ATCC Standards Development Organization (SDO) and in Capes-Davis et al., Match criteria for human cell line authentication: Where do we draw the line? Int J Cancer.2013;132(11):2510-9.

Add: Room 502, NO.2 Zhongxing Creative Park, Lane 1015, LongtengRoad, Songjiang District, Shanghai/ Tel:+86-021-33559493
E-mail: market@biowing.com

Test Results

1. STR profile

Table 2. STR and Amelogenin Genotyping Results of Cell line.

Loci	**Sample information**			**Cell Bank information**		
	Sample name：TPC-1			Cell line name：TPC-1		
	**Allele1**	**Allele2**	**Allele3**	**Allele1**	**Allele2**	**Allele3**
CSF1PO	11	12		11	12	
D3S1358	16	17		16	17	
D5S818	8	10		8	10	
D7S820	11	11		11	11	
D8S1179	11	17		11	17	
D13S317	11	12		11	12	
D16S539	9	9		9	9	
D18S51	13	16		13	16	
AMEL	X	X		X	X	
FGA	20	21				
TH01	9	9				
TPOX	11	11				
vWA	14	18				
D21S11	30	31.2				

Add: Room 502, NO.2 Zhongxing Creative Park, Lane 1015, LongtengRoad, Songjiang District, Shanghai/ Tel:+86-021-33559493
E-mail: market@biowing.com

2. database annotation

Figure 1. STR matching analysis

**Cell line name**	TPC-1
**Synonyms**	TPC1
**Accession**	CVCL_6298
**Resource Identification Initiative**	To cite this cell line use: TPC-1 (RRID:CVCL_6298)
**Comments**	Omics: Mitochondrial genome sequenced.<br>Omics: Transcriptome analysis.
**Sequence variations**	CCDC6-RET (RET/PTC1) gene fusion (PubMed=30737244).<br>Homozygous for CDKN2A p.Ala68fs (c.204_208delGGAGC) (PubMed=30737244).<br>Heterozygous for STAG2 p.Gln1089Ter (c.3265C>T) (PubMed=30737244).<br>Heterozygous for TERT c.228C>T (-124C>T); in promoter (PubMed=23833040; PubMed=30737244).
**Disease**	Thyroid gland papillary carcinoma (NCIt: C4035)<br>Differentiated thyroid carcinoma (ORDO: Orphanet_146)
**Species of origin**	Homo sapiens (Human) (NCBI Taxonomy: 9606)
**Hierarchy**	Children:<br>CVCL_6278 (BHP 10-3) CVCL_6283 (BHP 2-7) CVCL_6285 (BHP 7-13)<br>CVCL_9917 (FB-2) CVCL_V277 (RPTC-1)
**Sex of cell**	Female
**Age at sampling**	Adult
**Category**	Cancer cell line
**STR profile**	**Source(s):** PubMed=18713817; PubMed=21868764; PubMed=30737244<br><br>**Markers:**<br>Amelogenin: X<br>CSF1PO: 11,12<br>D3S1358: 16,17<br>D5S818: 8,10<br>D7S820: 11<br>D8S1179: 11,17<br>D13S317: 11,12<br>D16S539: 9<br>D18S51: 13,16<br>D21S11: 30,31.2<br>FGA: 20,21<br>TH01: 9<br>TPOX: 11<br>vWA: 14,18

Note: The STR online match analysis of the test cell against EXPASY database, showing cell number (Cell No.) and cell name.

3. Authentication

☐ The submitted sample profile is human, but not a match for any profile in the DSMZ STR database.

☑ The submitted profile is an exact match for the following human cell line(s) in the EXPASY STR database (8 core loci plus Amelogenin): **TPC-1** .

☐ The submitted profile is similar to the following DSMZ human cell line: /.

- **Note:** A cell line can be considered to be authenticated when 80% (exact match) of the alleles in its STR profile match profiles from tissue or other cell line samples from that donor or from database. Cell lines with between a 55% to 80% (similar) match require further profiling for investigation of relatedness.

Add: Room 502, NO.2 Zhongxing Creative Park, Lane 1015, LongtengRoad, Songjiang District, Shanghai/ Tel:+86-021-33559493
E-mail: market@biowing.com

Appendix:

1. Genotyping Strategy and Site Distribution

Table S1. Experimental Strategy and Sites

	Strategy 1	Strategy 2	Strategy 3	Strategy 4
1	D3S1358	D8S1179	D19S433	AMEL
2	VWA	D21S11	TH01	D1S1656
3	D7S820	D16S539	D13S317	D5S818
4	CSF1PO	D2S1338	TPOX	D12S391
5	PENTAE	PENTAD	D18S51	FGA
6			D6S1043	

The allele match algorithm compares the 8 core loci plus amelogenin only, even though alleles from all lociwill be reported when available.

2. DSMZ tools was used to carry on the cell line comparison, which contains 2455 cell lines STR data from ATCC, DSMZ, JCRB ,ECACC， GNE and RIKEN databases. If the cell is not included in the above cell library, users need to compared with other databases.

Technician: Jianan Zhang

Checked by: Ning Qian

Issued by: Yang Bai

Issue date: 3/30/2020

Add: Room 502, NO.2 Zhongxing Creative Park, Lane 1015, LongtengRoad, Songjiang District, Shanghai/ Tel:+86-021-33559493
E-mail: market@biowing.com
